# Modular Screening Reveals Driver Induced Additive Mechanisms of Baicalin and Jasminoidin on Cerebral Ischemia Therapy

**DOI:** 10.3389/fcvm.2022.813983

**Published:** 2022-02-21

**Authors:** Bing Li, Ying Wang, Hao Gu, Yanan Yu, Pengqian Wang, Jun Liu, Yingying Zhang, Yinying Chen, Qikai Niu, Bo Wang, Qiong Liu, Shuang Guan, Yanda Li, Huamin Zhang, Zhong Wang

**Affiliations:** ^1^Institute of Chinese Materia Medica, China Academy of Chinese Medical Sciences, Beijing, China; ^2^Institute of Basic Research in Clinical Medicine, China Academy of Chinese Medical Sciences, Beijing, China; ^3^College of Nursing, Chengde Medical University, Chengde, China; ^4^Dongzhimen Hospital, Beijing University of Chinese Medicine, Beijing, China; ^5^Guang'anmen Hospital, China Academy of Chinese Medical Sciences, Beijing, China

**Keywords:** combination therapy, synergistic effect, cerebral ischemia, network driver, modular pharmacology

## Abstract

Combination therapy with increased efficacy and reduced toxicity plays a crucial role in treating complex diseases, such as stroke, but it remains an insurmountable barrier to elucidate the mechanisms of synergistic effects. Here, we present a Driver-induced Modular Screening (DiMS) strategy integrated synergistic module and driver gene identification to elucidate the additive mechanisms of Baicalin (BA) and Jasminoidin (JA) on cerebral ischemia (CI) therapy. Based on anti-ischemia genomic networks BA, JA, and their combination (BJ), we obtained 4, 3, and 9 *On-modules* of BA, JA, and BJ by modular similarity analysis. Compared with the monotherapy groups, four additive modules (*Add-module*, BJ_Mod-4, 7, 9, and 13), 15 driver genes of BJ were identified by modular similarity and network control methods, and seven driver proteins (PAQR8, RhoA, EMC10, GGA2, VIPR1, FAM120A, and SEMA3F) were validated by animal experiments. The functional analysis found neuroprotective roles of the Add-modules and driver genes, such as the Neurotrophin signaling pathway and FoxO signaling pathway, which may reflect the additive mechanisms of BJ. Moreover, such a DiMS paradigm provides a new angle to explore the synergistic mechanisms of combination therapy and screen multi-targeted drugs for complex diseases.

## Introduction

Combination therapy with multiple targets has been clinically proved as a promising strategy to improve the treatment of complex diseases, such as stroke (1–3), but it remains an insurmountable barrier to elucidate the mechanisms of synergistic effects. For the polygenic pathological nature of cerebral ischemia (CI), it is unlikely to achieve optimal effects by intervening a single target, so the conventional single-gene or pathway-based paradigm may not systematically clarify the synergistic mechanism of combination therapy (4). To maintain the internal environment homeostasis, a biological system usually exists in a higher-order modular organization of networks (5, 6). It is, therefore, more adaptable to reveal the relationships between multi-targeted therapy and complex diseases from a modular targeted angle.

The module-based strategy integrates multi-omics data and has made much progress in decoding the systematic mechanism of drug effects (7, 8). Several studies have tried to identify the module biomarker or targets for cancer or many other complex diseases (9–11). The gene co-expression network at different conditions may manifest as intramodular edge rewiring and reflect the drug responses, and the allosteric On-modules can be identified (12). Functional synergistic modules related to inflammation-induced tumorigenesis were identified by integrating genome-wide gene prediction methods and CRISPR-Cas9 screening (13). A network-based methodology was proposed to identify drug combinations that can simultaneously modulate multiple targets within the same disease module, and aimed at achieving higher clinical efficacy and lower toxicity for specific diseases (14, 15). Moreover, a novel structural network controllability-based personalized driver genes and combinatorial drug identification algorithm (CPGD) was proposed to screen combinatorial drugs for an individual patient by targeting personalized driver genes from a network controllability perspective (16). Therefore, such module-targeted approaches may provide us with a more intensive understanding of the underlying mechanisms of drug synergistic actions from the “multiple-drugs, multiple-targets” perspective.

Baicalin (BA) and jasminoidin (JA) are bio-active ingredients extracted from Qingkailing injection, an effective preparation widely prescribed to treat ischemic stroke (17). Our previous studies showed that the combination of BA and JA (BJ) can significantly improve the therapeutic effect and exhibited an additive effect (18). It found that both BA and JA monotherapy exerted extensive pharmacological functions, such as neuroprotection, anti-inflammation, and anti-oxidation (19–21). Pathway-based studies demonstrated the cross-talks of BJ at both horizontal and vertical levels, such as apoptosis and survival, gamma-secretase activity, neurophysiological processes, development, reproduction, and regulation of lipid metabolism (22, 23). Our previous study explored the entropy-based divergent and convergent modular pattern to reveal the additive mechanisms of BJ, but the datasets and responsive module identification method were limited (24). The underlying additive mechanisms of BJ are far from clear at the systematic and genomic network level.

In this study, module similarity and network control methods were applied to identify BJ additive modules and driver genes based on anti-ischemic genomic networks. Through further functional analysis and experimental validation, the driver-induced modular screening (DiMS) strategy was used to systematically illuminate the additive mechanisms of BJ in CI treatment.

## Materials and Methods

### The Gene Expression Datasets of Different Drug Groups

The microarray gene expression datasets with 16,463 mice cDNA (Incyte Genomics, Inc., Santa Clara, CA, USA) were obtained from our previous study, which have been uploaded to ArrayExpress database (http://www.ebi.ac.uk/arrayexpress, E-TABM-662). A total of 105 adult male Kunming mice (12 weeks, weight range 38–48 g) were randomly divided into 5 groups (each consisting of 21 samples), nine mice from each group were selected to perform pharmacodynamics and gene expression examinations. The procedure of middle cerebral artery occlusion (MCAO) model preparation, drug administration, RNA isolation, and microarray preparation were described previously (22, 25). Five data groups were selected for analysis, such as the Sham group, Vehicle group (0.9% NaCl), BA group (baicalin, 5 mg/ml), JA group (jasminoidin, 25 mg/ml), and BJ group (combination of baicalin and jasminoidin at a ratio of 1:1).

To obtain the drug-induced differentially expressed genes (DEGs), one-way ANOVA and significance analysis of microarray were performed among the Sham, Vehicle, BA, JA, and BJ groups. Genes with a value of *p* < 0.05 and fold change >1.5 were screened out, and then Bonferroni correction was conducted to select the DEGs list of different drug groups for further analysis. To evaluate the relevance with CI of DEGs, known CI-related genes were collected from Disease Connect (http://disease-connect.org/), HPO (http://hpo.jax.org/), GeneCards (http://www.genecards.org/), and OMIM (https://omim.org/) database with “Cerebral Infarction” MeSH terms.

### Co-Expression Network Construction and Module Detection

Based on the DEGs of monotherapy (BA, JA) and combination (BJ) groups, gene co-expression networks were constructed by the weighted gene co-expression network analysis (WGCNA) algorithm (26). The similarity of each matrix of pairwise correlations among all pairs of probes across the measured samples was defined as Topological Overlap Matrices (TOM), and hierarchical clustering and Dynamic Hybrid Tree Cut algorithm (27) were applied to detect modules of each drug group. To get the optimal module partition quality, a cut-off threshold based on optimal average modularity *Q* (28) was selected, which was defined as:


$$\begin{array}{l} Q=\frac{1}{2n}\underset{ij}{\sum }\left[a_{ij}-\frac{k_{i}k_{j}}{2n}\right]\sigma \left(c_{i},c_{j}\right)\; \end{array}$$

where *n* is the number of edges in a module, *a*_*ij*_ is the similarity between gene *i* and gene *j*, and *k*_*i*_ is the connectivity of gene *i*, σ(*c*_*i*_, *c*_*j*_) = 1 only when gene *i* and *j* are in the identical module. All the detected modules were named by the order number and visualized by Cytoscape software (29).

To test the robustness of module identification results, WGCNA was performed across all 3 drug groups, and Z_summary_ statistic (30) was used to check the preservation of module in the BA, JA, and BJ groups. Compared with particular groups, module with a Z_summary_ ≥ 2 indicates its preservation (30).

### The Drug-Responsive On-Modules and Additive Modules Identification

To character the mechanisms of action of BA, JA, and BJ, the drug-responsive co-expression module other than a single gene was identified by modular differential analysis. By comparing all module pairs from condition-specific networks, we can get a similarity matrix *B* between the *i*-th module (*A*_*i*_) from the network *N*_1_ and *j*-th module (*A*_*j*_) from the network *N*_2_, and the Jaccard index was used to evaluate the similarity of *B*_*ij*_ and identify the differentially expressed sub-networks as conserved or condition specific modules which may be associated with different drug effects (31). Two threshold values of *min*(*s*)+θ_1_ and *max*(*s*)-θ_2_ were defined to select the condition conserved and specific modules, where *s* indicates to what extent the *i*-th module might be affected by certain conditions (28). In our analysis, we set θ_1_ and θ_2_ as 0.1.


$$\begin{array}{l} B_{ij}=\frac{N_{1}\left(A_{i}\right)\cap N_{1}\left(A_{j}\right)}{N_{1}\left(A_{i}\right)\cup N_{2}\left(A_{j}\right)}\; \end{array}$$

Taken the Vehicle group as the background network, module in the drug group >*max*(*s*)-θ_2_ was considered as a conserved module (CM), < *min*(*s*)+θ_1_ was considered as a drug-responsive module, i.e., the On-module. Similarly, compared with the monotherapy (BA, JA) group, the shared and specific On-modules of BJ could be found, and the specific On-modules of BJ were considered as the additive module (Add-module).

### Identification of Driver Genes of the Additive Modules

To further find the hub genes which may contribute greater to the synergistic effect, we compared the BJ On-module genes distribution in BA and JA, the characteristic genes for different drug groups were obtained. Compared with BA and JA respectively, a personalized network control algorithm (PNC) was used to identify the personalized driver genes of additive modules from a network controllability perspective (32). The network control method aims to find a minimum set of driver nodes that steer the condition-specific networks to the desired control objectives, such as the synergistic effect. So, based on the genetic data of different drug treatments, a structure-based network control model of the PNC package (16) was applied to identify the driver genes of the BJ Add-module. The intersection of driver nodes compared with BA and JA were selected as the synergistic driver genes of BJ. To validate the controllability of driver genes, the NetworkX Python package was used to calculate node importance of the BJ Add-module's network, such as degree centrality, eigenvector, betweenness, PageRank, and clossness.

### Functional Annotation of Additive Modules

To characterize the function of Add-modules, we performed Gene ontology (GO) and Kyoto Encyclopedia of Genes and Genomes (KEGG) pathway enrichment analysis by using the ClusterProfiler R package (V3.16.0) (33, 34). We selected Gene Set Enrichment Analysis (GSEA) method and set organism to be “Homo Species.” For each Add-module, all the gene names were transferred from “symbol” to uniformed “entrezid,” and a modified Fisher's exact *p* with Benjamini–Hochberg (BH) adjustment method was used to select the enriched GO terms or KEGG pathways, terms with the *p* < 0.05 were considered as significant. The representative pathway was illustrated by KEGG, and the driver genes involved in a particular pathway were highlighted.

### Animal Model and 2, 3, 5-Triphenyltetrazolium Chloride (TTC) Staining

To illustrate the role of Add-modules, western blotting (WB) experiments were conducted to validate representative driver gene-encoded proteins' expression patterns in different groups. Thirty-five SD rats were divided into Sham, Vehicle, BA, JA, and BJ groups, and the MCAO model, and drug administration methods were consisted with our previous studies (22, 25). Longa's score (5 points) was used to assess the neurological deficit changes score for rats in each group. The hippocampus tissue of 5 rats from each group was removed from the brains at 24 h Post-ischemia, of which 2 rats in each group were used to calculate the infarct ratio by TTC staining. The infarct area was determined by using the Image-Pro Plus 6.0 software.

### Western Blotting

After protein extraction and cell lysis, standard WB operation was performed. In brief, tissue total proteins (40 mg) were loaded on 10% sodium dodecyl sulfate–polyacrylamide gel electrophoresis (SDS-PAGE) gels and transferred onto a nitrocellulose membrane. The membrane was blocked using 5% skim milk for 2 h, then incubated overnight with primary antibodies at 4°C. The membrane was then incubated with a secondary antibody, such as anti-RhoA (1:5,000 dilution, Abcam, ab187027), anti-EMC10 (1:500 dilution, Solarbio, K009317P), anti-SEMA3F (1:2,000 dilution, Affinity, DF8611), anti-VIPR1 (1:1,000 dilution, Affinity, DF5172), anti-PAQR8 (1:1,000 dilution, Novus, NBP2-92893), anti-GGA2 (1:1,000 dilution, Proteintech, 10356-1-AP), and β-actin (1:1,000 dilution, Boster, BM0627) as an internal control. The band density was measured by BandScan 5.0. Each protein measurement was taken in three replicates.

## Results

### The DEGs of Each Group With Efficacy Variation

Our prior studies have found that BA, JA, and BJ can significantly reduce the ischemic infarct volume compared with the Vehicle group, and BJ was more effective than BA or JA monotherapies, which demonstrated the additive pharmacological effect of BJ in CI treatment (18).

**Figure 1:**
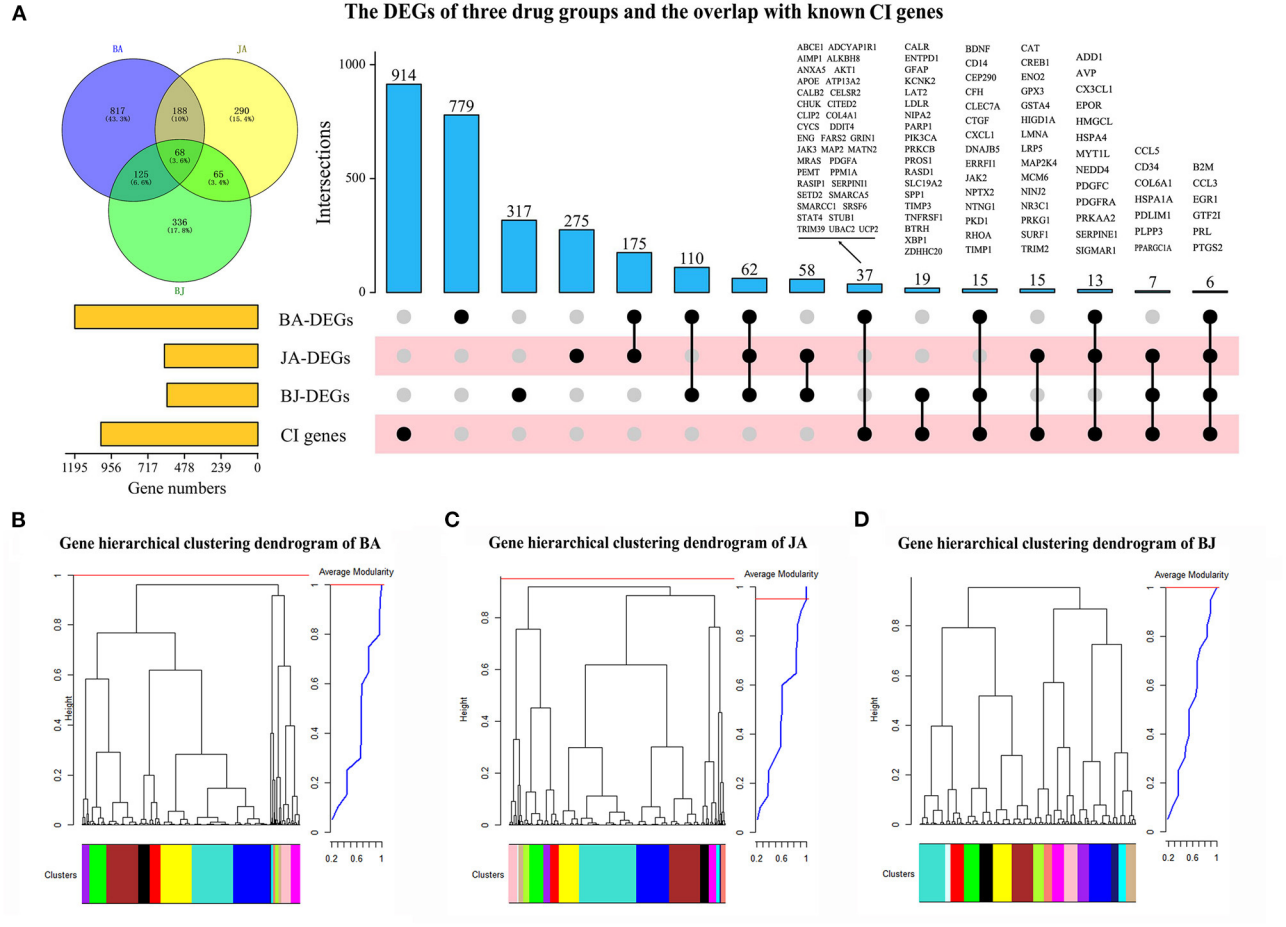
The differentially expressed genes (DEGs) and co-expression modules of three-drug groups. **(A)** The number of DEGs for Baicalin (BA), Jasminoidin (JA), and their combination BJ, and their overlapped genes compared with known cerebral ischemia (CI) related genes. **(B–D)** Hierarchical clustering dendrogram of BA, JA, and BJ, the cluster tree branches labeled by colors were represented modules which were obtained by Dynamic Tree Cut algorithm with maximum average modularity.

Based on the expression profiles of 16,463 mice cDNA, 1,208, 614, and 600 DEGs were obtained in BA, JA, and BJ, respectively (Supplementary Table 1). There are 68 (3.6%) shared DEGs among the three drug groups, and 188 (10%), 126 (6.6%), 65 (3.4%) overlapped DEGs between BA and JA, BJ and BA, BJ and JA, respectively (Figure 1A). For the synergistic combination BJ group, there are 336 unique DEGs.

Compared with the collected 1,026 known CI-related genes (Supplementary Table 1), a total of 112 (10.9%) DEGs were overlapped, of which 47 were involved in the BJ group (Figure 1A, Supplementary Table 2). Six known CI-related genes (B2M, CCL3, EGR1, GTF2I, PRL, and PTGS2) were differentially expressed in all of the three drug groups, and 19 CI-related genes were BJ specific (Figure 1A).

### Co-Expression Modules in the Three Drug Groups

Based on the expression profiles of DEGs in BA, JA, and BJ (Supplementary Tables 3–5), we use the WGCNA package (26) to construct the weighted gene co-expression networks and identify modules for different drugs. Topological overlap matrices (TOM) and Dynamic Hybrid Tree Cut algorithm were used to perform average linkage hierarchical clustering and partition the branches of dendrogram as modules (27). A module is defined as closely connected sub-network which may involve common biological functions, the maximal modularity was selected as the optimal cut-off threshold for the module identification (28). We identified 15, 16, and 16 co-expression modules for BA, JA, and BJ, respectively. Each module corresponded to a branch of the resulting clustering tree which was labeled by a unique color (Figures 1B–D). The detailed composition of module gene labeled by color and number of each group is shown in Supplementary Tables 3–5.

To test the robustness of module identification results, we identified modules across all 3 drug groups by combining the DEGs. A total of 16 modules were obtained based on the combined dataset. Except one module, all the other modules had Z_summary_ ≥2 compared with BA, JA, and BJ, which indicates the preservation and robustness of these modules (Supplementary Figures 4A,B).

### The Conserved and On-Modules of BA, JA, and BJ

**Figure 2:**
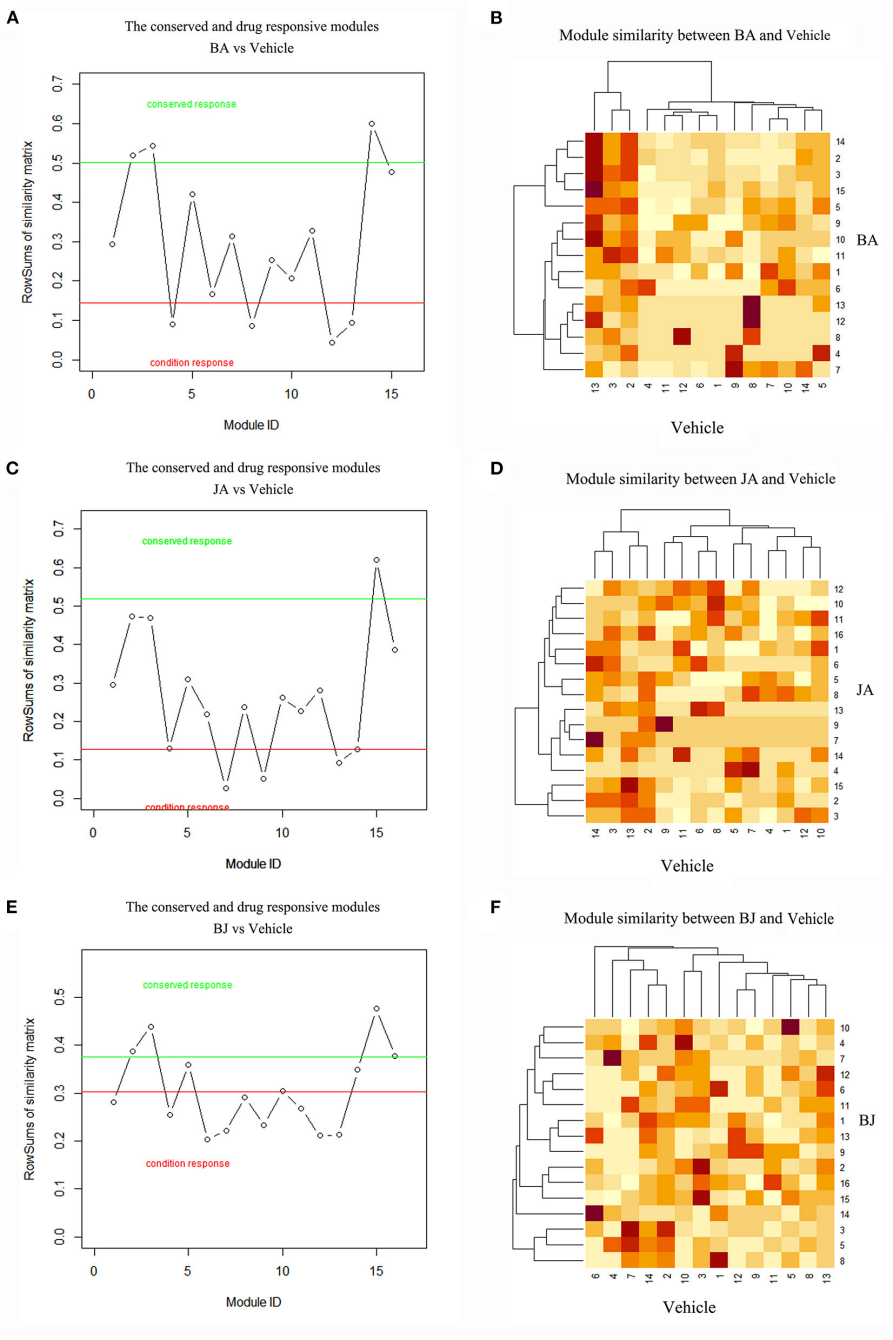
The On-modules and conserved modules of three-drug groups compared with Vehicle. **(A,B)** BA vs. Vehicle group, the red line represents the threshold of max(s)-θ2 and green line represents the threshold of min(s)+θ1, modules under the red line were BA's On-modules, and modules above the green line were BA's conserved modules. The heatmap showed the similarities between BA and Vehicle modules. **(C,D)** JA vs. Vehicle group. **(E,F)** BJ vs. Vehicle group.

Cerebral ischemic was a polygenetic disease, it is more informative to identify modules that are conserved or activated across certain conditions. Compared with the Vehicle group, we identify the conserved and drug-responsive modules by modular similarity and Jaccard index, and modules less than the minimum similarity cutoff value were considered as *On-modules*. By comparing all module pairs of drug groups to the Vehicle group, we got 4 (*BA_Mod4, 8, 12, 13)*, 3 (*JA_Mod7, 9, 13*), and 9 (*BJ_Mod1, 4, 6, 7, 8, 9, 11, 12, 13*) *On-modules* of BA, JA, and BJ, respectively. BJ had the most responsive On-modules. Compared with the Vehicle group, there were 3(*BA_Mod2, 3, 14*), 1(*JA_Mod15*), and 3 (*BJ_Mod2, 3, 15*) conserved modules of BA, JA, and BJ, respectively. The detailed conserved and *On-modules* as well as their similarities are shown in Figures 2A–F.

### The Additive Modules of BJ

**Figure 3:**
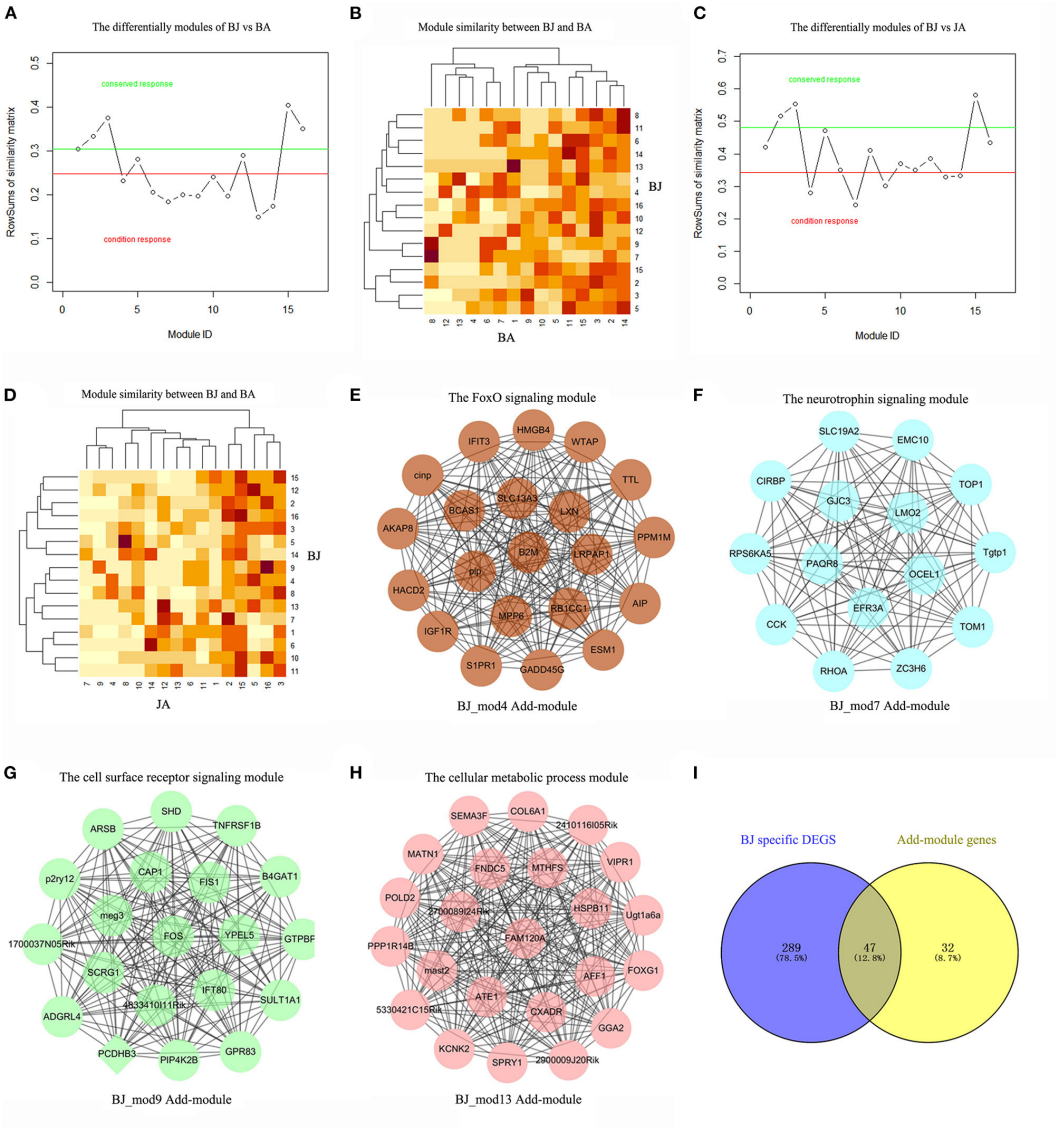
The Add-modules of BJ compared with BA and JA. **(A)** The BJ specific On-modules compared with BA, modules under the red line were BJ's specific modules. **(B)** The heatmap of module similarities between BJ and BA. **(C)** The BJ specific On-modules compared with JA. **(D)** The heatmap of module similarities between BJ and JA. **(E–H)** The visualization of BJ Add-modules, which are the specific On-modules compared with both Vehicle group, BA and JA. Each module was named by the ID and its enriched functions. **(I)** The genes' overlapping between BJ Add-modules and BJ specific DEGs.

Similarly, on comparing BJ On-modules with BA and JA respectively, we got the characteristic modules that were responsible only to the synergistic BJ group, i.e., the Add-modules. As shown in Figures 3A–D, a total of 4 Add-modules (*BJ_Mod4, 7, 9, 13*) were found. These modules were composed of 21, 15, 19, and 23 genes, respectively, which are visualized in Figures 3E–H. Among the 79 genes in the BJ Add-modules, forty-seven genes (59%) were overlapped with BJ unique DEGs, and their Jaccard coefficient is 0.128 (Figure 3I). Besides, three BJ modules (*BJ_Mod2, 3, 15*) were conserved at all the pairwise condition comparisons.

### The Driver Genes of BJ Additive Modules

**Figure 4:**
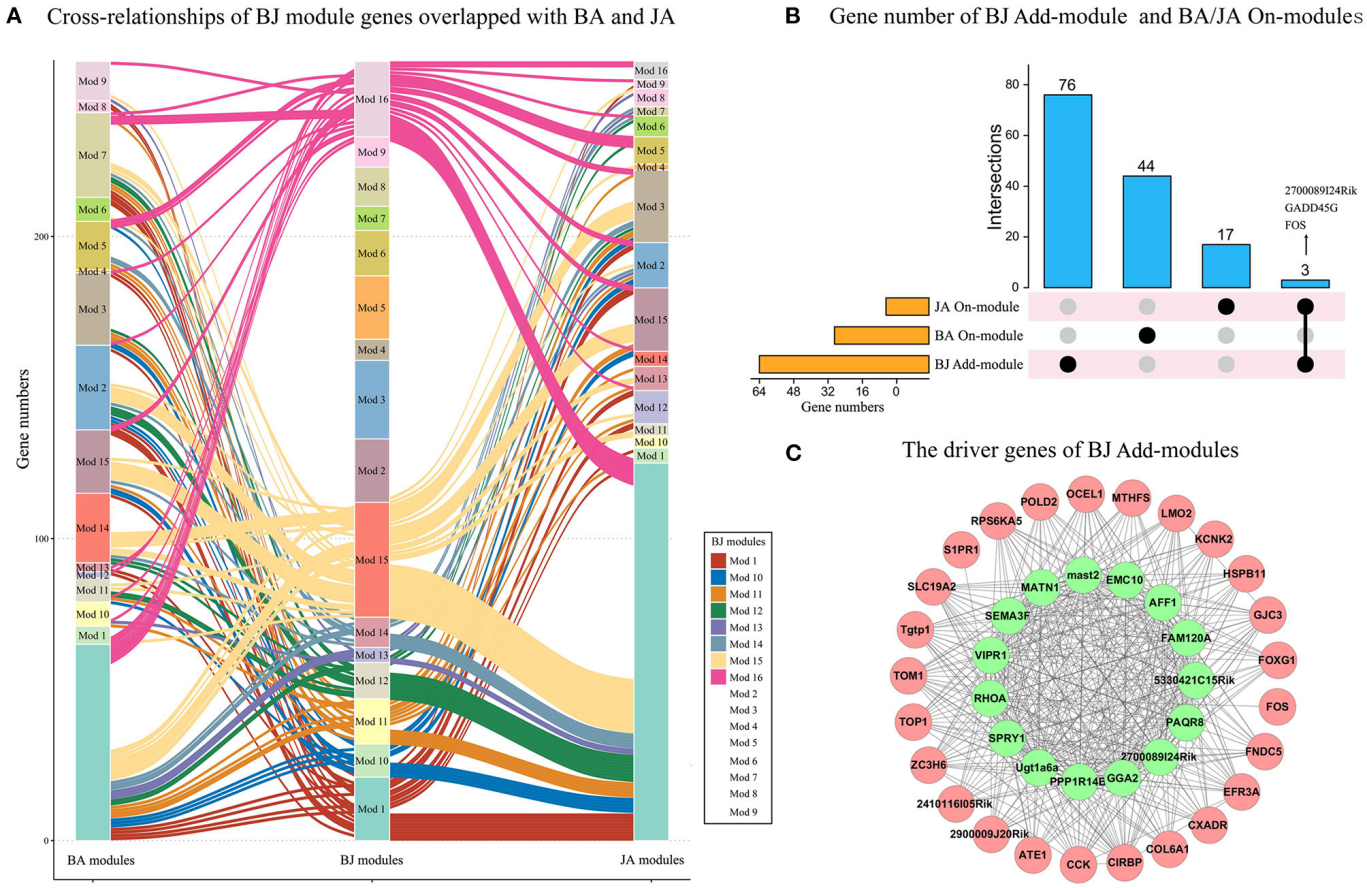
The driver genes of BJ Add-modules. **(A)** The BJ module genes' cross-relationship compared with BA and JA. **(B)** The BJ Add-module genes' overlap with BA and JA On-modules. **(C)** The driver genes (green nodes) of BJ Add-modules.

To identify the specific driver genes of BJ, which may control the whole network and catalyze the synergistic effect, we first compare the BJ module genes' distribution in the other two sets of modules. Compared with BA and JA modules, eight BJ modules had no overlapped genes, such as the *BJ_Mod4, BJ_Mod7*, and *BJ_Mod9* Add-modules (Figure 4A). With regard to the On-modules, only 3 overlapped genes in Add-modules were shared with JA On-module, i.e., *FOS, GADD45G*, and *2700089I24Rik* (Figure 4B). These may explain the specificity of BJ Add-modules. Furthermore, fifteen driver genes (GGA2, 2700089I24Rik, PAQR8, 5330421C15RIK, FAM120A, AFF1, EMC10, MAST2, PPP1R14B, MATN1, SEMA3F, VIPR1, SPRY1, RHOA, and UGT1A6A) were identified based on minimum control principle (Figure 4C).

**Figure 5:**
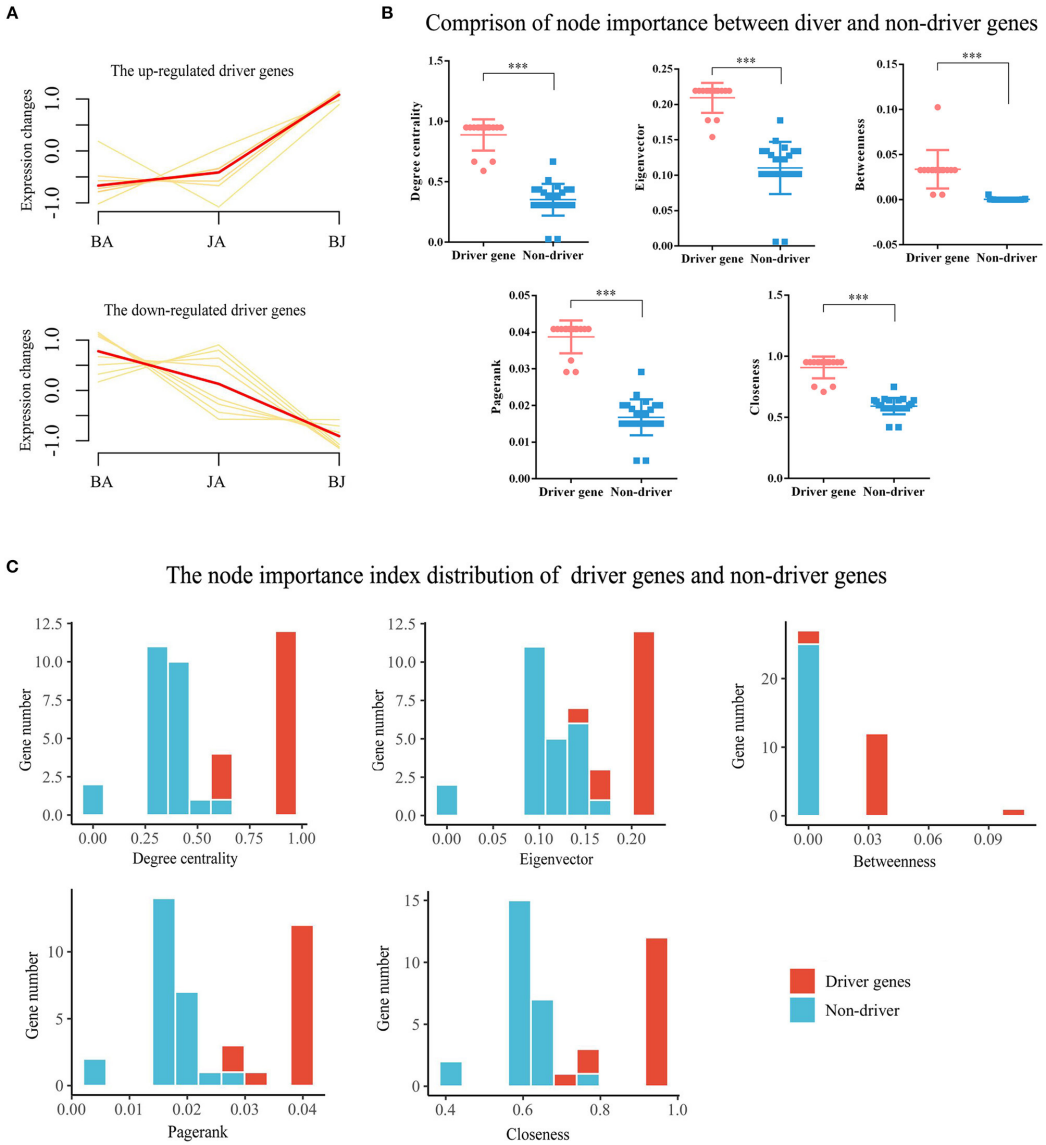
Validation the roles of driver genes. **(A)** The expression changes of driver genes in the three-drug groups. **(B)** The comparison of driver genes and non-driver genes on 5 node importance indicators, ***p < 0.001. **(C)** The decile interval distribution of driver and non-driver genes based on the network node importance indicators of degree centrality, eigenvector, betweenness, PageRank, and clossness.

Furthermore, the expression changes and 5 node importance indicators were calculated to validate the essential role of driver genes. Compared with BA and JA groups, the driver genes were significantly upregulated or downregulated (Figure 5A). On the degree centrality, eigenvector, betweenness, PageRank, and closeness node importance indicators, the driver genes were more important than non-driver genes (Figure 5B). From the decile interval distribution of BJ Add-module genes, we can see that almost all the driver genes have a higher value on the 5 node importance indicators (Figure 5C).

### Significant Biological Functions of Additive Modules

**Figure 6:**
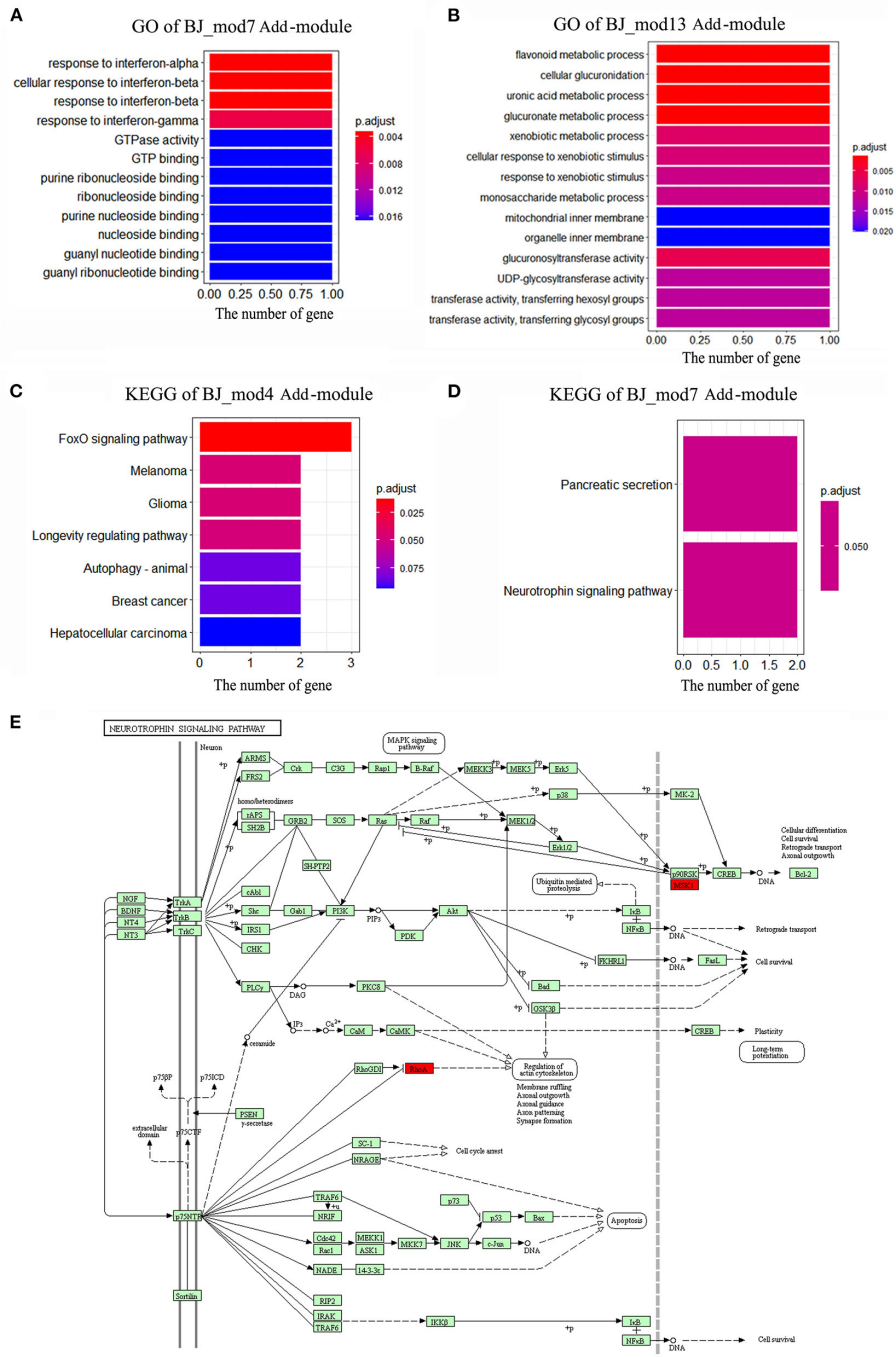
The enriched functions of BJ Add-modules. **(A)** The significant Gene ontology (GO) terms enriched by BJ-Mod 7. **(B)** The significant GO terms enriched by BJ-Mod 13. **(C)** The significant Kyoto Encyclopedia of Genes and Genomes (KEGG) pathways enriched by BJ-Mod 4. **(D)** The significant KEGG pathways enriched by BJ-Mod 7. **(E)** The representative Neurotrophin signaling pathway enriched by BJ-Mod 7, and the driver genes of BJ are labeled as red.

Based on the BJ Add-modules, GO function and KEGG pathway enrichment analysis were performed, and the top significant GO terms and pathways are shown in Figures 6A–D. The results showed that the genes in *BJ_Mod4* are mainly enriched in the FoxO signaling pathway, Melanoma, Glioma, Longevity regulating pathway, Autophagy, etc. The *BJ_Mod7* is enriched in Pancreatic secretion, Neurotrophin signaling pathway and response to interferon, GTPase activity, GTP binding, nucleoside binding GO terms, etc. In the Neurotrophin signaling pathway, BJ can activate the *MSK1* and *RhoA* driver genes, which promotes the cell survival and axonal outgrowth (Figure 6E). *BJ_Mod13* is mainly enriched in flavonoid, glucuronate, uronic acid, xenobiotic metabolic process, and mitochondrial inner membrane, etc. Several enriched functions are closely related to neural functional recovery which may reflect the synergistic mechanisms of BJ in CI treatment. Based on the functional enrichment results, we defined the functions of each Add-modules (Figures 3E–H).

### The Protein Expression of BJ Additive Module's Driver Genes

To validate the essential roles of Add-modules in contribution to the additive effect of BJ, MCAO rats and WB were used to examine the expression patterns of driver proteins in different groups. TTC staining showed that the infarct volume was increased in the Vehicle group compared with the Sham group, and significantly decreased in the three drug groups in comparison with the Vehicle group (Supplementary Figures 1A,B). As shown in Supplementary Figure 1C, the neurological function score of three drug groups were significantly decreased compared with the Vehicle group (*p* < 0.001), and the decline was more significant in BJ in comparison with BA and JA (*p* < 0.01).

**Figure 7:**
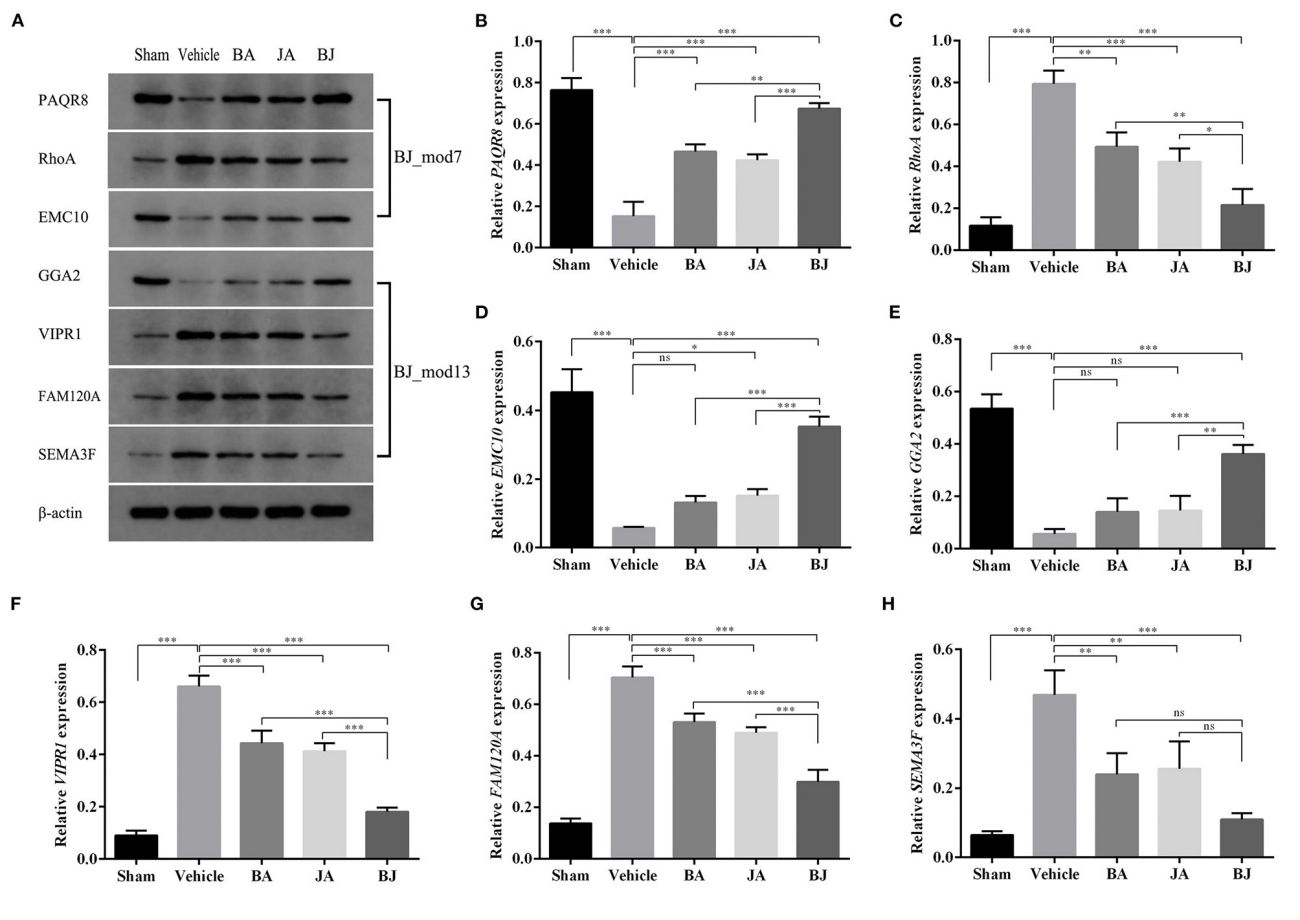
Validation of the driver proteins' expression level in each group. **(A)** The band diagrams of 7 representative driver proteins and β-actin in each group. **(B–H)** The expression level comparison among groups for PAQR8, RhoA, EMC10, GGA2, VIPR1, and FAM120A, SEMA3F determined by western blot. *p < 0.05, **p < 0.01, ***p < 0.001, and ns, non-significant.

Seven driver proteins (PAQR8, RhoA, EMC10, GGA2, VIPR1, FAM120A, and SEMA3F) from two Add-modules (*BJ_Mod7* and *BJ_Mod13*) were selected for WB analysis, the results are shown in Figure 7. Except for EMC10 in BA and GGA2 in BA and JA, all the driver proteins in three-drug groups were significantly changed in the expression level compared with the Vehicle group (3 upregulate and 4 downregulated). Furthermore, except for SEMA3F, the expression levels of other proteins in BJ were changed significantly more than that in BA and JA.

## Discussion

In view of the complexity of diseases and the multi-target effect of drugs, conventional “one target, one pathway” studies could not fully illuminate the synergistic mechanism of drug combination therapy (35, 36). There is an increasing evidence that both pathogeneses of diseases and mechanism of action of drugs have a modular basis, as genes or proteins may interact with each other to execute certain functions in biological networks (5, 37). Targeting functional modules which are composed of multiple closely connected targets would be more reasonable to systematically reveal the multi-targeted or synergistic effects of drugs in the context of network (38, 39). In this study, based on the gene expression data of anti-CI model treated by BA, JA, and BJ, we systematically identified 4 additive modules and 15 driver genes of BJ by modular analysis and network control methods, which may reveal the synergistic mechanism of BJ in the treatment of CI from systematic and network perspective. Moreover, such a modular targeted research paradigm may provide an alternative approach to explore the synergistic mechanisms of drug combination therapy and therapeutic target screening for complex diseases.

Modularity is a fundamental property of biology and disease networks (5). Several studies have attempted to identify the module biomarker for complex diseases or drug targets (9–11). Under different drugs treatment, the gene co-expression relationship could be changed and manifest as the intramodular rewiring, so we can identify the modular targets of drugs based on the condition of specific modular configuration (40). Based on the functional enrichment analysis, we found that the 4 identified BJ Add-modules were closely associated with CI. For example, the *BJ_Mod4* enriched FoxO signaling pathway plays an important role in the activation of neuroprotection against transient global CI (41, 42). Activating the *BJ_Mod7* enriched Neurotrophin signaling pathway can influence nerve terminals to promote neuronal survival and axonal growth by regulating the expression of essential proteins (43, 44). The *BJ_Mod7* is also enriched Pancreatic secretion pathway, while regulation of Post-ischemic glucose plays an important role in suppressing the neuronal damage for CI therapy (45, 46). The response to interferon-beta, interferon-gamma and GTP binding GO terms are both closely related to the protective effect against ischemic stroke (47–49).

In biological networks, controlling a minimum set of driver nodes can steer the networks to desired control objectives, such as the synergistic effect. We found 15 driver genes of BJ Add-modules by network control approach, and seven of them (PAQR8, RhoA, EMC10, GGA2, VIPR1, FAM120A, and SEMA3F*)* were validated by animal experiments. Previous studies found that Rho-kinase including RhoA exhibited effect on neurovascular damage and might be potential therapeutic target in the treatment of CI (50, 51). SEMA3F is expressed in layers adjacent to cortical interneuron migratory streams as well as in the striatum, suggesting that they may have a role in guiding these cells throughout their journey (52). Progestin and AdipoQ receptor (PAQR) family, such as PAQR8, regulates a wide range of cognitive, neuroendocrine, neuroimmune, and neuroprotective functions (53). FAM120A (also known as C9orf10) is a RNA-binding protein that may protect cells from oxidative stress-induced apoptosis by the activation of SFKs (54). The study suggests that SPRY1 contributes to the regulation of CNS functions by influencing both neuronal differentiation under normal physiological processes and neuronal survival under pathological conditions (55). Other protein, such as VIPR1, has an important role in neuroprotection (56). These demonstrated the effectiveness of Add-modules and its driver genes in elucidating the synergistic mechanism of BJ.

In conclusion, the present DiMS strategy effectively-identified 4 targeted additive modules and 15 driver genes of BJ based on anti-ischemia genomic networks, functional enrichment, and animal experiments further validated its additive mechanism. Moreover, such a modular targeted research paradigm may provide a new angle to explore the synergistic mechanisms of combination therapy and screen multi-targeted for complex diseases.

## Data Availability Statement

Publicly available datasets were analyzed in this study. This data can be found at: https://www.ebi.ac.uk/arrayexpress/, E-TABM-662.

## Ethics Statement

The animal study was reviewed and approved by the Ethics Review Committee for Animal Experimentation, China Academy of Chinese Medical Sciences.

## Author Contributions

ZW and BL contributed to the conceptualization of the manuscript. BL, HG, YY, PW, BW, and QN performed the methodology. YW, HG, QL, SG, QN, and YL are responsible for the investigation. BL and YZ contributed to the visualization. ZW and HZ performed the supervision. YW, HG, BL, and YC contributed to the writing—original draft. ZW, HZ, and JL contributed to writing, reviewing, and editing. All authors contributed to the article and approved the submitted version.

## Funding

This work was funded by the CACMS Innovation Fund (No. CI2021A05052 to BL), the National Natural Science Foundation of China (No. 81803966 to BL and No. 81904064 to PW), the Open Research Fund of Chengdu University of Traditional Chinese Medicine Key Laboratory of Systematic Research of Distinctive Chinese Medicine Resources in Southwest China (No. 2019GZ2011011 to ZW), and the Fundamental Research Funds for the Central Public Welfare Research Institutes (ZXKT21024 to BL and ZZ13-YQ-078 to HG).

## Conflict of Interest

The authors declare that the research was conducted in the absence of any commercial or financial relationships that could be construed as a potential conflict of interest.

## Publisher's Note

All claims expressed in this article are solely those of the authors and do not necessarily represent those of their affiliated organizations, or those of the publisher, the editors and the reviewers. Any product that may be evaluated in this article, or claim that may be made by its manufacturer, is not guaranteed or endorsed by the publisher.

## Supplementary Material

Supplementary Figure 1 The infarction volume and neurological function evaluation of each group at 24 h after MCAO. **(A)** Representative triphenyltetrazolium chloride (TTC) staining images of each group. **(B)** The infarction volume of each group. **(C)** The neurological deficit score comparison among each group, ^**^*p* < 0.01, ^***^*p* < 0.001.

Supplementary Figure 2 The uncropped images of the western blots presented in Figure 7.

Supplementary Figure 3 The raw films of the western blots results.

Supplementary Figure 4 The network modules for the combined dataset across all the 3 groups. **(A)** The hierarchical cluster tree of the combined dataset, and modules are labeled with colors below the dendrogram. **(B)** The Z_summary_ value of the 16 modules. Module with Z_summary_ ≥2 indicates its preservation. The green and blue dotted lines indicate the boundary of strong and moderate preservation cutoff.

Supplementary Table 1 The differentially expressed genes (DEGs) of three-drug groups and cerebral ischemia (CI) related genes.

Supplementary Table 2 The intersections among DEGs of three-drug groups and CI-related genes.

Supplementary Table 3 The expression level of DEGs in Baicalin (BA) group and its corresponding modules.

Supplementary Table 4 The expression level of DEGs in Jasminoidin (JA) group and its corresponding modules.

Supplementary Table 5 The expression level of DEGs in BJ (combination of baicalin and jasminoidin at a ratio of 1:1) group and its corresponding modules.

Supplementary Data Sheet 1 The relevant code/script used in this study.
