# Supplementary figures and images for: Modular Screening Reveals Driver Induced Additive Mechanisms of Baicalin and Jasminoidin on Cerebral Ischemia Therapy
Source: Front Cardiovasc Med. 2022 Feb 21;9:813983. doi: 10.3389/fcvm.2022.813983 (PMC8899124; doi:10.3389/fcvm.2022.813983)

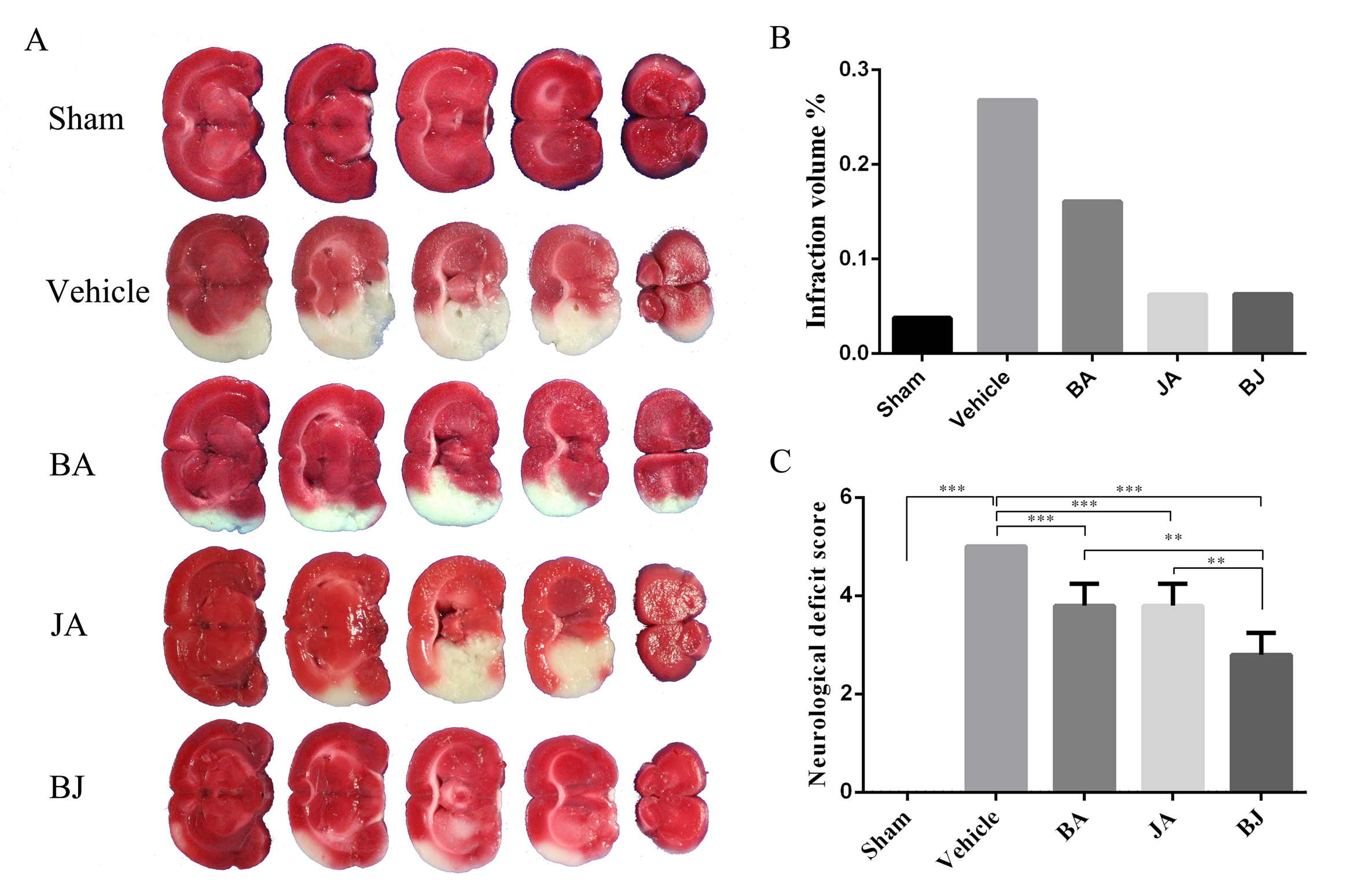

Supplement: Supplementary Figure 1 — The infarction volume and neurological function evaluation of each group at 24 h after MCAO. (A) Representative triphenyltetrazolium chloride (TTC) staining images of each group. (B) The infarction volume of each group. (C) The neurological deficit score comparison among each group, **p < 0.01, ***p < 0.001. [file Image_1.TIF]

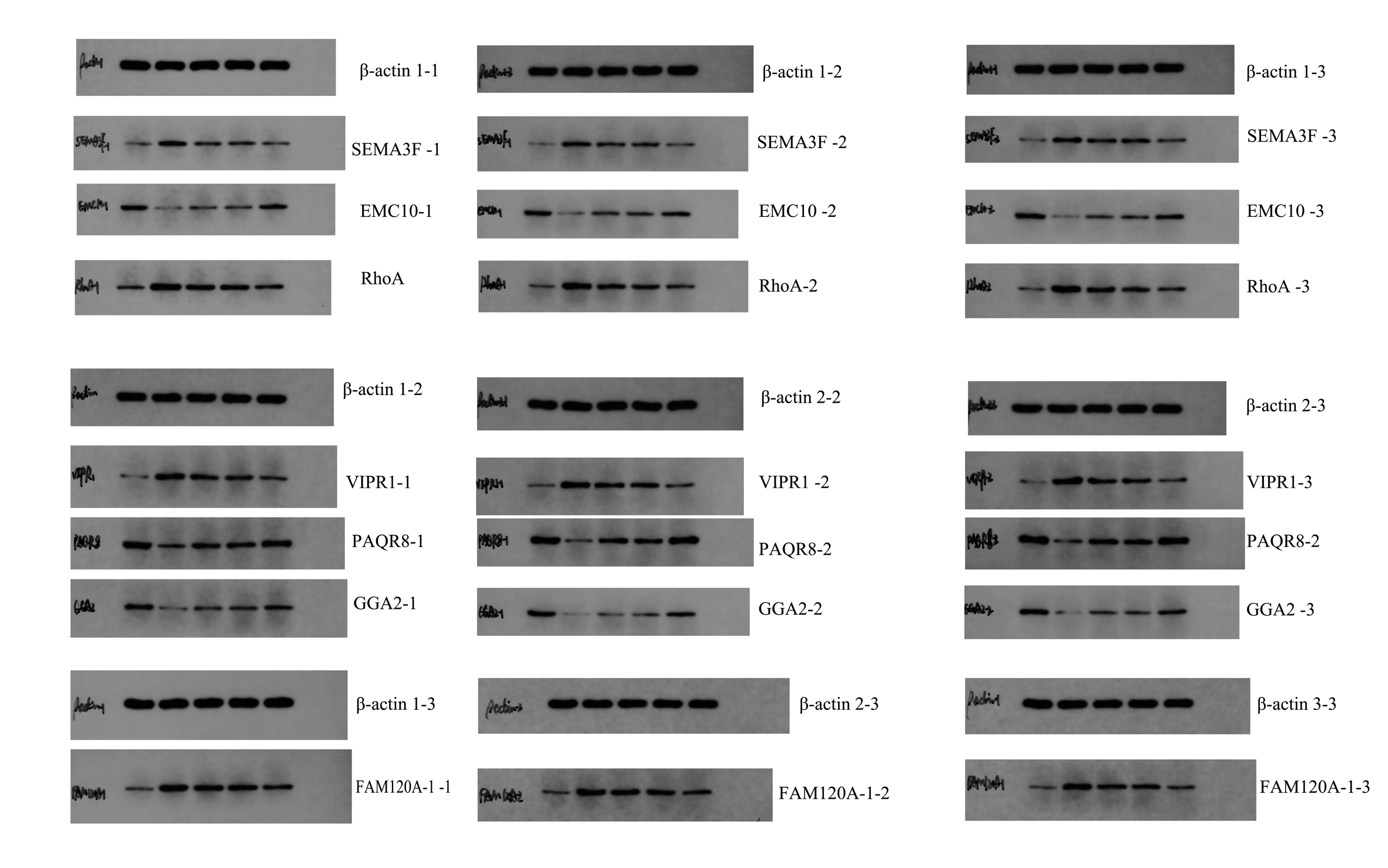

Supplement: Supplementary Figure 2 — The uncropped images of the western blots presented in Figure 7. [file Image_2.TIF]

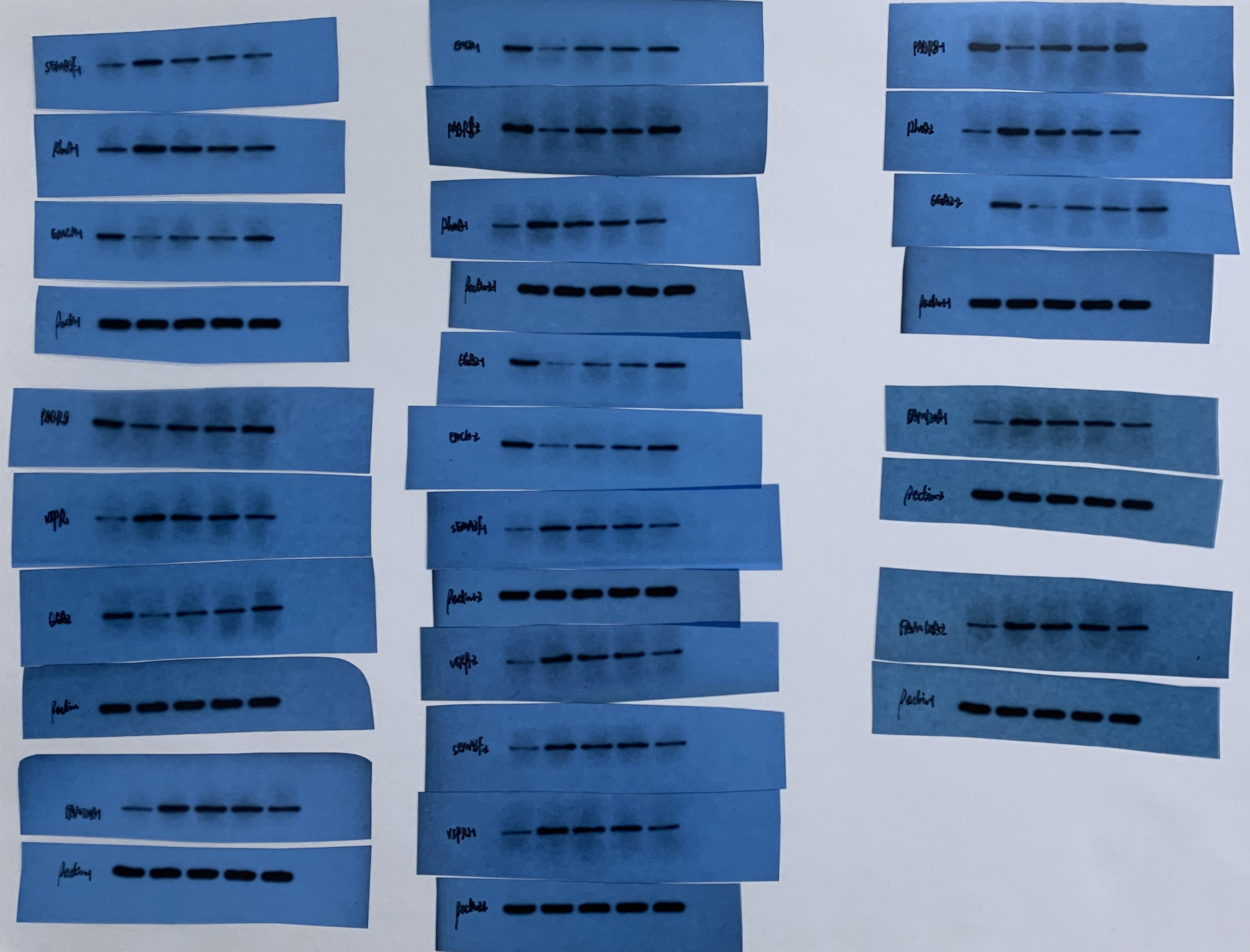

Supplement: Supplementary Figure 3 — The raw films of the western blots results. [file Image_3.JPEG]

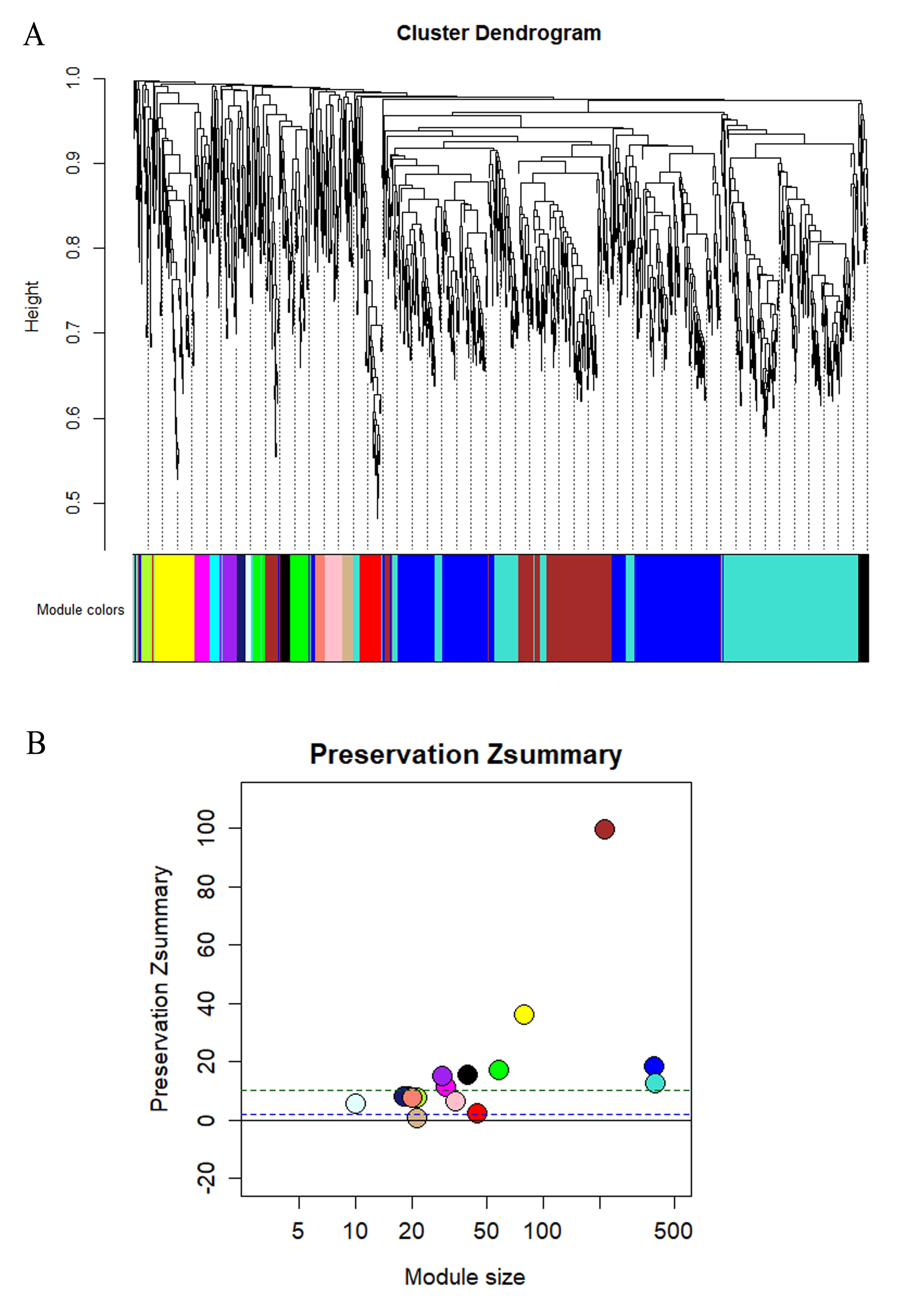

Supplement: Supplementary Figure 4 — The network modules for the combined dataset across all the 3 groups. (A) The hierarchical cluster tree of the combined dataset, and modules are labeled with colors below the dendrogram. (B) The Zsummary value of the 16 modules. Module with Zsummary ≥2 indicates its preservation. The green and blue dotted lines indicate the boundary of strong and moderate preservation cutoff. [file Image_4.JPEG]
