# Supplementary material for: Modular Screening Reveals Driver Induced Additive Mechanisms of Baicalin and Jasminoidin on Cerebral Ischemia Therapy
Source: Front Cardiovasc Med. 2022 Feb 21;9:813983. doi: 10.3389/fcvm.2022.813983 (PMC8899124; doi:10.3389/fcvm.2022.813983)
Supplement: Supplementary Data Sheet 1 — The relevant code/script used in this study. [file Data_Sheet_1.docx]

Additional File 1

The relevant code/script used in this study

1. **The R code for module similarity comparison (for Fig1.B-D, Fig2 and Fig3.A-D)**

> library(MODA)

> library(WGCNA)

# read the drug group data

dat0=read.csv("BJ-3sam.csv", header=TRUE)

names(dat0)

datSummary=dat0[,c(1,5:8)]

datExprFemale <- t(dat0[,2:4])

no.samples <- dim(datExprFemale)[[1]]

dim(datExprFemale)

# Set the columns names to probe names

colnames(datExprFemale) = datSummary$NameID

# This module assignment was obtained by libing

# read the vehicle group data

data=read.csv("sham-3sam.csv", header=TRUE)

datExprMale = t(data[,2:4])

colnames(datExprMale) = data$NameID

dim(datExprMale)

ResultFolder = 'D:/QKL-big/qkl-big'

# set the CuttingCriterion

CuttingCriterion = 'Modularity' # could be Density or Modularity

# make the pairwise comparative analysis

indicator1 = 'BJ' # indicator for data profile 1

indicator2 = 'sham' # indicator for data profile 2

specificTheta = 0.1 #threshold to define condition specific modules

conservedTheta = 0.1#threshold to define conserved modules

intModules1=WeightedModulePartitionHierarchical(datExprFemale, ResultFolder, indicator1,CuttingCriterion)

intModules2 <- WeightedModulePartitionHierarchical(datExprMale, ResultFolder, indicator2,CuttingCriterion)

CompareAllNets(ResultFolder, intModules1,indicator1,intModules2,indicator2,

specificTheta,conservedTheta)

# get the similarity matrix and heat map

JaccardMatrix <- comparemodulestwonets(ResultFolder,intModules1,intModules2, paste('/DenseModuleGene_',indicator1,sep=''), paste('/DenseModuleGene_',indicator2,sep=''))

1. **The Matlab code for PNC driver genes identification (for Fig4.C )**

clc

clear

% $Id: main_PNC.m Created at 2019-05-29 22:22:20 $

% by Weifeng Guo, Northwestern Polytechtical University, China

% Copyright (c) 2014-2019 by Key Laboratory of Information Fusion Technology of Ministry of Education in Northwestern Polytechnical University,

% and key Laboratory of Systems Biology in Shanghai Institutes for Biological Science;

% If any problem,pleasse contact shaonianweifeng@126.com for help.

%Remainder: Please install gurobi before running our code

%Remainder: Please install gurobi before running our code

%Remainder: Please install gurobi before running our code

%**************Part 1:Input the information of samples and network information****

%**************sample information**************

%Example: BJ compared to BA

expression_BJ_fileName = 'BJ-all.txt';

expression_BA_fileName = 'BA-all.txt';

%%**************Part 2:Network control methods output****

[ PNC_driver_result ] = PNC( expression_BJ_fileName,expression_BA_fileName);

%%**************Part 3:save the result****

save PNC_driver_result

function [ PNC_driver_result ] = PNC( expression_BJ_fileName,expression_BA_fileName)

%we output the sample-specific driver profiles by using different control

%methods

% Input:

% expression_fileName including expression_BJ_fileName and expression_BA_fileName)

% index:denotes we use which network construction method

% Output:

% The sample-specific driver profiles of PNC;

% The column is the samples and the rows is the genes. The value 鈥?鈥?denoted that the gene is driver genes;

%************************part1:LOAD sample data and network data************************

%********************obtain the paired expression data******************

expression_BJ_fileName = 'Example_BJ.txt';

expression_BA_fileName = 'Example_BA.txt';

[BJ,~,name_BJ]=importdata(expression_BJ_fileName);

gene_list=BJ.textdata(2:end,1);BJ_data=BJ.data;

[BA,~,name_BA]=importdata(expression_BA_fileName);

Sample_name_BA=BA.textdata(1,2:end);BA_data=BA.data;

data=BJ_data;ref_data=BA_data;

function [ new_x,index ] = Opti_weight_nc( dz,NN )

%non-lineaqr controllability of undirected networks

% input:

% z:network structure

% scores:the scores of each node

% lamda:the parameter of our PDC

% Output:

% lc_index:the number of driver nodes

%***********************solve the problem**************************************

%**********************MATLAB2014******************************

uz=unique(dz);

[ind,z1]=ismember(dz(:,1),uz);

[ind,z2]=ismember(dz(:,2),uz);

z=[z1 z2];

N=max(max(z));

lamda=0;

%N=max(max(z));

scores=ones(N,1);

function [ index_R,p ] = SSN( sample,ref )

%function:construct the SSN

% Input:

% sample:calculated sample

% ref:the reference samples

% Output:

% adjacency_matrix:the network structure

%a example

% sample=new_T(:,1);

% ref=new_N;

[R,P]=corrcoef(ref');

final_R0=R;

final_R0(isnan(final_R0))=0;

NEW_data=[ref sample];

[R1,P1]=corrcoef(NEW_data');

final_R1=R1;

final_R1(isnan(final_R1))=0;

index_R=final_R1-final_R0;

[m,n]=size(ref);

Z=index_R./((1-final_R0.^2)/(n-1));

Z(Z==inf)=max(max(Z));

Z(Z==-inf)=-max(max(Z));

Z(isnan(Z))=0;

clear NEW_data final_R1 final_R0 R0 R1 P P1

p=1-normcdf(abs(Z));

end

1. **The python code of node importance calculation for driver genes (for Fig5 )**

Import the BJ add-module networkx as nx

#get network

def getNodeEdgesfromfile(file_path_name):

nodes_list = []

edges_list = []

with open(file_path_name) as fpn:

for line in fpn:

lines = str(line).split(',')

if lines[0].strip() not in nodes_list:

nodes_list.append(lines[0].strip())

if lines[1].strip() not in nodes_list:

nodes_list.append(lines[1].strip())

if lines[0].strip() != lines[1].strip():

edge = (lines[0].strip(),lines[1].strip())

if edge not in edges_list:

edges_list.append(edge)

return nodes_list,edges_list

#calculate the node importance indexes

def getNodeimpotance(G):

degree = nx.degree(G)

degree_rs = nx.degree_centrality(G)

pagerank_rs = nx.pagerank(G)

eigenvector_rs = nx.eigenvector_centrality(G)

closeness_rs = nx.closeness_centrality(G)

betweenness_rs = nx.betweenness_centrality(G)

return degree,degree_rs,pagerank_rs,eigenvector_rs,closeness_rs,betweenness_rs

if __name__=="__main__":

filepath = "D:\\network_ctm\\"

filename = "BJ-Umodcoex095-and-PPI-network.csv"

nodes_list,edges_list = getNodeEdgesfromfile(filepath + filename)

G = nx.Graph()

G.add_edges_from(edges_list)

degree,degree_rs,pagerank_rs,eigenvector_rs,closeness_rs,betweenness_rs = getNodeimpotance(G)

file_wr = "file_wr2.csv"

with open(filepath + file_wr, "a") as fw:

for i in G.nodes():

fw.write(str(i))

fw.write(",")

fw.write(str(degree[str(i)]))

fw.write(",")

fw.write(str(degree_rs[str(i)]))

fw.write(",")

fw.write(str(pagerank_rs[str(i)]))

fw.write(",")

fw.write(str(eigenvector_rs[str(i)]))

fw.write(",")

fw.write(str(closeness_rs[str(i)]))

fw.write(",")

fw.write(str(betweenness_rs[str(i)]))

fw.write("\n")

fw.flush()

fw.close()
